# Biotic and abiotic effects on density, body size, sex ratio, and survival in immature stages of the European woodwasp, *Sirex noctilio*

**DOI:** 10.1002/ece3.6966

**Published:** 2020-11-03

**Authors:** Jeff R. Garnas, Katie E. Vann, Brett P. Hurley

**Affiliations:** ^1^ Department of Natural Resources and the Environment (NREN) University of New Hampshire Durham NH USA; ^2^ Department of Zoology and Entomology University of Pretoria Pretoria South Africa; ^3^ Forestry and Agricultural Biotechnology Institute (FABI) University of Pretoria Pretoria South Africa; ^4^ Present address: Weyerhaeuser Weyerhaeuser NR Vanceboro NC USA

**Keywords:** community drivers, *Pissodes*, population dynamics, Siricidae, Woodwasps

## Abstract

Resource quality can have direct or indirect effects on female oviposition choice, offspring growth and survival, and ultimately on body size and sex ratio. We examined these patterns in *Sirex noctilio* Fabricus, the globally invasive European pine woodwasp, in South African *Pinus patula* plantations. We studied how tree position as well as natural variation in biotic and abiotic factors influenced sex‐specific density, larval size, tunnel length, male proportion, and survival across development. Twenty infested trees divided into top, middle, and bottom sections were sampled at three time points during larval development. We measured moisture content, bluestain fungal colonization, and co‐occurring insect density and counted, measured, and sexed all immature wasps. A subset of larval tunnels was measured to assess tunnel length and resource use efficiency (tunnel length as a function of immature wasp size). Wasp density increased from the bottoms to the tops of trees for both males and females. However, the largest individuals and the longest tunnels were found in bottom sections. Male bias was strong (~10:1) and likewise differed among sections, with the highest proportion in the middle and top sections. Sex ratios became more strongly male biased due to high female mortality, especially in top and middle sections. Biotic and abiotic factors such as colonization by *Diplodia sapinea*, weevil (*Pissodes* sp.) density, and wood moisture explained modest residual variation in our primary mixed effects models (0%–22%). These findings contribute to a more comprehensive understanding of sex‐specific resource quality for *S. noctilio* and of how variation in key biotic and abiotic factors can influence body size, sex ratio, and survival in this economically important woodwasp.

## Graphical abstract

Many insects exhibit large variation in body size and sex ratio, though specific mechanisms driving such variation are often elusive. We examined a suite of potential biotic and abiotic drivers of this variation in invasive European woodwasp (*Sirex noctilio*) across larval development stages in two South Africa pine plantations. We found that position within the tree, and to a lesser degree log moisture, *Pissodes* weevil densities, and *Diplodia sapinea* bluestain fungal colonization influenced wasp densities, growth rates, and survivial in gender‐specific ways. The trends uncovered have potentially important consequences of the ecology and management of this species.

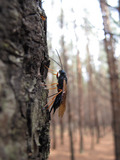

## 1 INTRODUCTION

Body size and sex ratio are two traits with important implications for population growth rates in insects (Chown & Gaston, 2010; Foelker & Hofstetter, 2014; Price, 2002). Clinal, interpopulation, and individual variation in body size are among the most studied aspects of the ecology of insects, in part due to a clear relationship with fitness. Fecundity (Honěk, 1993), survival (Ovadia et al., 2007), flight and associated behaviors (i.e., foraging, oviposition, and dispersal; Bruzzone et al., 2009; Gaudon et al., 2016), mating success (Benelli et al., 2016), generation time/voltinism, and longevity (Flatt et al., 2014) often strongly covary with body size, though sometimes in complex ways. In addition, body size has both basic and applied relevance for its relationships with temperature, latitude, host quality, nutrition, and ecological niche, making it an attractive target of investigation.

Variation in sex ratio has also been a major focus of both theoretical and empirical studies and likewise has enormous practical implications for the growth of populations. Body size and sex ratio are also linked in insects via sexual dimorphism, where larger females are typically more costly to produce and/or must grow more in a similar time frame or else feed longer or on higher quality food (Foelker & Hofstetter, 2014; Ovadia et al., 2007). Further, investment in smaller males may be favored in resource‐poor environments (Craig et al., 1992).

Both body size and sex ratio have been implicated in the success of invasive species, though no universal patterns appear to exist. For example, small body size is frequently associated with both crypsis and rapid population growth rates (Savage et al., 2004), traits clearly associated with introduction and spread in insects. In established populations, selection may favor larger females which confers higher fecundity and in some cases enhanced flight and therefore dispersal capacity (Bruzzone et al., 2009; Hajek et al., 2017). However, stabilizing selection and/or sexual dimorphism is evident where large size equates to reduced agility (e.g., limiting male success in mating swarms) or increased predation risk (Blanckenhorn, 2000; Neems et al., 1998). Female bias or even parthenogenesis may be positively associated with invasion success in some taxa (Garnas et al., 2016) and is a favored trait among intentionally introduced biocontrol agents since only females contribute to establishment, spread, and ultimately control. Despite theoretical expectations of sex ratio parity or female bias, including among nonsocial haplodiploid species (where males arise from unfertilized, haploid eggs and females from diploid, fertilized eggs; Hamilton, 1967), male bias does occur in some insect species or populations (Wrensch & Ebbert, 1993) and is predicted under local resource competition (Silk, 1984).

**FIGURE 1:**
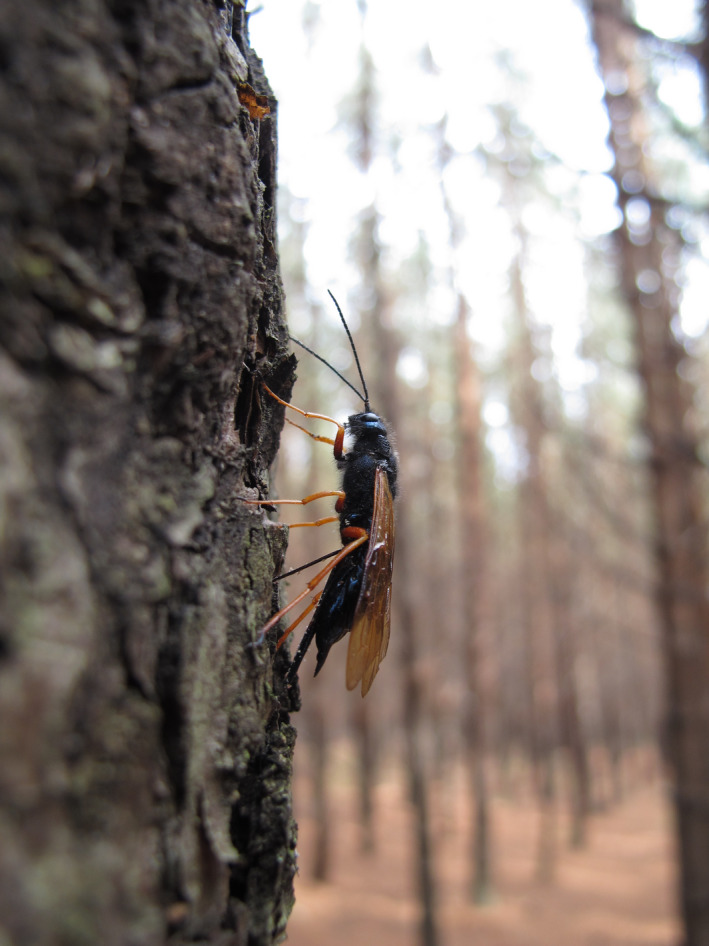
Female *Sirex noctilio* ovipositing on a *Pinus patula* tree in the Mpumalanga Province, South Africa. Photograph credit: Jeff Garnas

The European woodwasp, *Sirex noctilio* Fabricus, is a globally invasive species now present on all continents where pine occurs as a native or exotic (Boissin et al., 2012; Slippers et al., 2012; Figure 1). Native to North Africa and Eurasia, the species is characterized by low density populations that utilize primarily suppressed or highly stressed trees as hosts, mostly in the genus *Pinus* (Chrystal, 1928; Madden, 1968; Spradbery & Kirk, 1978). Spruce is also attacked but is less preferred. In the Southern Hemisphere, this wasp is a major threat to plantation pine forestry where trees are planted at high density, particularly when grown for pulp. In South Africa, *S. noctilio* was first detected in the Cape Province in 2004 infesting Monterey pine (*Pinus radiata* D. Don), but quickly spread throughout the country and now causes the most damage in high‐density pulp stands of *Pinus patula* Schiede ex Schltdl. & Cham. (Mexican weeping pine) in the country's interior (Lantschner et al., 2014). Despite the implementation of a multi‐tiered biological control effort using the ibaliid wasp *Ibalia leucospoides* Hochenwarth and aggressive annual inundative releases of *Deladenus siricidicola* Bedding, a mycetophagous nematode and reproductive parasite of *S. noctilio*, silvicultural and other management approaches are still required (Hurley, Croft, et al., 2012).

**FIGURE A1:**
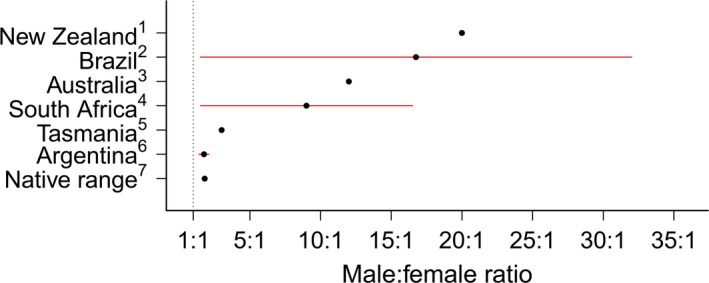
Mean (black dot) and range (red lines) male:female ratio as reported in the *S. noctilio* literature. Dotted line denotes a 1:1 ratio. ^1^Spradbery and Kirk (1978); ^2^Eskiviski et al. (2004); ^3^Neumann et al., (1987); ^4^Taylor (1981); ^5^Hurley et al. (2008); ^6^Iede et al. (1998); ^7^Zondag and Nuttall (1977)

Among the most striking aspects of the biology of *S. noctilio* is the enormous variation in body size and sex ratio. Like many insects, *S. noctilio* is size dimorphic, with females averaging 24.1 mm (5th [95th] quantile = 14 [38] mm) and males averaging 18.8 mm (5th [95th] quantile = 10 [31] mm) from head to tip of the abdomen (Hurley et al., unpublished data). However, even when accounting for sex, size variation in *S. noctilio* exceeds that of other insects across seven orders specifically identified for their size variability in a recent review (Gouws et al., 2011). Sex ratio is likewise highly variable in *S. noctilio* and often strongly male biased, particularly in some invasive populations (Figure A1). Broad surveys across the native range in Europe estimated the *S. noctilio* male:female ratio at 1.75:1 (Spradbery & Kirk, 1978). A more recent study found a 2.8:1 M:F ratio in Spain (Lombardero et al., 2016). Such levels of male bias are not uncommon for haplodiploid species, including wasps, though unfortunately, few studies have assessed variation in this ratio over space and/or time. In the introduced range, estimates range from approximately equivalent to those in its native range (x̄ = 1.75:1; range = [1.4,2.1]) up to 20:1 or even 32:1 in New Zealand and Brazil, respectively (Iede et al., 1998; Zondag & Nuttall, 1977), with estimates of 10:1 or greater being common (Figure A1).

Host resource quality for *S. noctilio* may be influenced by various abiotic and biotic factors, including nutrient content or availability of food resources (Thompson et al., 2013), moisture content (Morgan, 1968), and defensive chemistry (Coutts & Dolezal, 1966). These and other factors may act directly on wasp oviposition choice and larval development. Indirect effects of resource quality on larval development are also possible. For example, female *S. noctilio* obligately carry a mutualist fungus, the basidiomycete *Amylostereum areolatum* (Chaillet ex Fr.) Boidin, that they inject into oviposition sites (King, 1966). While the functional role of this fungus is not precisely known, it does not appear to act as a direct food source but may function as an external gut by supplying key digestive enzymes for lignocellulosic breakdown as well as sterols (Thompson et al., 2014). Indeed, larvae do not ingest xylem directly but appear to use extracted sugars as an energy source for microbial nitrogen fixation (Thompson, 2013). Variation within trees that influences the growth of *A. areolatum* or that favors or deters its microbial competitors (most prominently the sapstaining, or "bluestain" fungi; Awmack & Leather, 2002; Haavik et al., 2015, 2019; Hurley, Hatting, et al., 2012; Klepzig et al., 2004; Krams et al., 2012) could impact wasp growth and survival. In South Africa, *Diplodia sapinea* (endophytic latent pathogen and causal agent of tip blight) and several bark‐beetle vectored species of the *Leptographium* and *Ophiostoma* cause characteristic staining of pine xylem (Bihon et al., 2011; Zhou et al., 2001). These fungi aggressively colonize wood within declining trees and so are prominent potential antagonists to *S. noctilio* and/or *A. areolatum*.

In this study, we examined how natural variation in biotic and abiotic factors influences larval and pupal densities, body size, tunneling behavior, and resource use efficiency across sites, trees, log sections, and sampling dates (early, middle, and late with respect to wasp development). We examined these questions using structured environmental sampling and extensive dissections of *S. noctilio*‐infested logs across two *P. patula* plantations in eastern South Africa and at three heights. Our findings contribute to a better understanding of the role of biotic and abiotic factors in driving *S. noctilio* population dynamics allowing improved predictions of future patterns of abundance of this invasive species.

## 2 MATERIALS AND METHODS

### 2.1 Tree and log sampling

We collected logs from two *P. patula* pulp plantations in Mpumalanga, South Africa (Table 1a), where moderate to high densities of *S. noctilio*‐infested trees were known to be present. To locate trees infested by *S. noctilio*, we used the symptoms known to correlate with infestation, specifically the presence of resin drops and yellow or red foliage color (Dodds et al., 2010; Talbot, 1977). At three time points throughout the *S. noctilio* larval life cycle, ten random *S. noctilio‐*infested *P. patula* trees were selected from each of the two sites. In the sampled area, wasp adults emerge and lay eggs from late October/early November and sampling dates were selected to represent the early (March 2012), late (September 2012), and mid (June 2013) developmental stages. Since the mid‐development larval stage was taken from a different larval cohort, only the early and late stages were used to estimate sex‐specific patterns in survival and size distribution. Each main stem was visually divided into thirds, and a 90 cm section was cut from the approximate middle of each third to represent the bottom, middle, and top of each tree. Trees that showed no evidence of active tunneling on the cross‐sectional face (top or bottom) of at least one cut log section were discarded and a new tree selected. Diameters of the top and bottom of each log were measured and wood volume calculated using mean log diameter, assuming a perfect cylinder. Four moisture measurements from each log were taken immediately after felling using a Delmhorst RDM‐3 moisture probe (species setting: *P. radiata* [*P. patula* was not available]) and averaged to represent log moisture. To prevent wasp development prior to log dissection, logs were stored at 4°C at the Forestry and Agricultural Biotechnology Institute (FABI), University of Pretoria.

**TABLE 1:** Summary of four‐part modeling for each of the five univariate response variables considered in the core analyses implemented in this research

(a) All dates model		Values and/or Notes	
Univariate response variables	Count, all immatures, dm^−3^ (ln + 1 transformed)	Range = 0–51.9; x̄ [*SD*] = 5.0 [5.7]
	Head width (mm)	0.05–5.1; 2.2 [0.6]
	Male proportion	0–1; 0.84 [0.18]
	Tunnel length (mm)	0.9–29.5; 8.5 [3.8]
	Resource use efficiency (RUE)	Residuals after regressing larval/pupal head width by tunnel length
Predictor variables, fixed effect plus interactions	Sampling date	Early, middle, late
	Site	Two *Pinus patala* pulp plantations, ages 12 and 14 years, planting density (2 × 3 m grid) = 1,650 stems/ha
	Position	Log position (Bottom, middle, top)
	Sex	Male or female
	Sampling date × Site	Two‐way interactions
	Sampling date × Position	
	Sampling date × Sex	
	Site × Sex	
	Position × Sex	
	Sampling date × Site × Position	Select three‐way interactions
	Sampling date × Site × Sex	
	Sampling date × Position × Sex	
Random effects	Tree (Sampling date, Site)	Tree nested within Sampling date and Site, plus two‐way interactions with Position and Sex
	Position × Tree (Sampling date, Site)	
	Sex × Tree (Sampling date, Site)	

(b) Residual models, other predictors		Values and/or Notes
Univariate responses	Studentized residuals from each of the univariate models in (a) above	—
Additional predictors	Live wasps (dm^−3^ of xylem)	Range = 0–51.9; x̄ [*SD*] = 5.0 [5.7]
	Dead wasps (dm^−3^ of xylem)	0–9.8; 0.7 [1.3]
	Cross‐sectional bluestain (%)	0–89.9; 7.3 [12.9]
	*Pissodes* sp. (dm^−3^ of xylem)	0–22.0; 1.2 [3.0]
	Log diameter (cm)	6.7–13.7; 10.5 [1.5]
	Standardized log diam. (z‐score)	−1.2 to 1.1; 0.05 [0.8]
	Log moisture (%)	5–60, 24.2 [12.7]
	*D. siricidicola* parasitism	NA (none found)

(c) Immature stage models, late sampling dates only		(d) Immature stage models, late sampling dates only
Univariate responses	Same as (a)	Same as (b), with residuals saved from models outlined in (c)
Fixed effects	Same as (a) but with “Immature stage” substituted for “Sampling date”	Same as (b)
Random effects	Tree (Site)	—
	Position × Tree (Site)	
	Sex × Tree (Site)	

Each response variable was examined in two ways using linear mixed models: (1) across sampling dates (i.e., including sampling date as a predictor) with larvae and pupae pooled together as “immatures” (a); and (2) late stage only (where pupae uniquely occurred) removing “Sampling date” and including “Immature stage” as a predictor (c). Models (b) and (d) use as their response variables the studentized residuals from (a) and (c), respectively, to examine the explanatory power of additional biotic or abiotic “latent” variables (measured at the log scale) not explained by base models a and c.

### 2.2 Tunnel sampling and larval extraction

Using a hand‐operated hydraulic MAC‐AFRIC™ 10‐ton hydraulic log splitter, logs were carefully split into small fragments (~200 mm long by 5 mm wide) to extract all *S. noctilio* larvae and pupae. Up to eight randomly selected tunnels per log were meticulously traced back to their origin at the phloem–xylem interface and measured for length and maximum width. Only tunnels within the middle two thirds of log length (60 cm) were selected for measurement to exclude tunnels that continued outside the sampled area and so could not be fully measured. Tunnels were measured as they were uncovered during splitting using digital calipers; lengths were summed across split fragments to provide a total length for each selected gallery. All measured tunnels were separated by at least 10 cm throughout their length. All larvae and pupae were removed from the log, counted, measured, and sexed. Larval sex was determined by the presence of hypopleural organs in cuticular folds on either side of female larvae between the first and second abdominal segments (Gilmour, 1965). Also called mycetangia (reflecting their function in collection and storage of arthrospores of the fungal symbiont, *A. areolatum*), these organs are absent in the first instar. Since all larvae encountered in this study were second instar or greater, this did not represent a source of error. Pupal sex was determined by the presence or absence of an ovipositor, visible through the pupal skin. Measurements included length (from the tip of the head capsule to the tip of the tail spine at the end of the abdomen), head capsule width, and wet and dry body mass. In addition, ten larvae per log section were randomly selected and dissected to confirm the presence or absence of the parasitic nematode, *D. siricidicola* (Bedding & Akhurst, 1974) used as a biocontrol agent in South Africa and elsewhere. Lastly, the number of dead larvae as well as identity and abundance of other insects in the log sections were recorded.

### 2.3 Additional predictors

Tree and log‐level variables (Table 1b) were considered as possible predictors of multiple response factors including larval density, head width, tunnel length, resource use efficiency (RUE), and male proportion. RUE was calculated as the residuals from a linear regression between larval/pupal head width as the response variable and tunnel length as the predictor. In this way, RUE is standardized across tunnel lengths resulting in negative values when larval size was lower than expected based on the volume of wood excavated and positive values when *S. noctilio* size was higher than expected. To estimate mean proportion of each log section colonized by bluestain fungi, both ends of each log section were photographed and the cross‐sectional area stained percentage calculated using ImageJ (Igathinathane et al., 2008; Sheffield, 2007). Given that by far the most dominant sapstaining fungus of pine in this region is *D. sapinea* (Fr.) Fuckel (Bihon et al., 2011), we selected a random subsample of five bluestained logs per sampling date to isolate and confirm fungal identities. Small (1–2 mm) plugs of mycelia were collected with a sharp, sterile scalpel and placed on a Petri dish containing 2% malt extract agar and serially replated to remove contaminants. To eliminate potential strain diversity, single hyphal tips from each culture were extracted using a sterile needle and replated after being allowed to grow for 10–14 days. Ultimately, viable cultures were obtained from ten logs, from which DNA was extracted, amplified and Sanger sequenced using the fungal‐specific primers ITS1F and ITS4 (Gardes & Bruns, 1993). All isolates were confirmed as *D. sapinea*. In addition, we collected and identified all insects cocolonizing the bark or wood tissue with *S. noctilio*.

### 2.4 Data analysis

We constructed univariate linear mixed effects for wasp density, head width, male proportion, tunnel length, and RUE. For each response variable, two separate models were considered (Table 1). The first suite of models examined patterns across sampling dates, pooling larvae and pupae together as immatures. These models were constructed as follows: sampling date, site, log position (herein “position”), and sex plus all two‐way and select three three‐way interactions (sampling date × position ×sex, sampling date × site × sex, and sampling date × sex × position) were included as fixed effects in the mixed model design (Table 1a, c). Random effects included tree (sampling date, site) plus the interaction between tree (sampling date, site) and sex and position. The second suite of models focused only on the late sampling date where both larvae and pupae were present, and immature stage (larva or pupa) was considered as an additional predictor. No attempt to categorize larval instar was made. These models were identical to those already described but with immature stage (again, larva vs. pupa) substituted for sampling date in the fixed effects component. Here, random effects were tree(site) and its two‐way interactions with position, sex, and stage. To conform to the assumption of our models (approximate normality and homogeneity of variance), we log + 1 transformed the wasp density response variable. No other variables required transformation to meet model assumptions.

Since not all of the of the log sections sampled contained wasp larvae, we could not calculate larval size, tunnel volume, and related parameters for all logs, leading to an unbalanced design. Since restricted maximum likelihood analysis (REML) can be sensitive to lack of balance (Garson, 2012), we randomly selected the appropriate number of trees so as to achieve a balanced design with respect to each response variable tested. However, since a total of 23 log sections did not contain wasps, and since these were distributed randomly across sites, trees, and sampling dates, this would have led to considerable reductions to our dataset. To evaluate bias correction methods for unbalanced, nested designs, we created twenty independent, random, fully balanced subsets of our data and compared model results of each of these with the full, unbalanced dataset. All nested mixed models were performed in JMP 15.0 (SAS Institute, Cary, NC) with appropriate Satterthwaite corrections to degrees of freedom and associated *F* tests. Comparisons of balanced and unbalanced models are reported in the Results section for head width, but ultimately our simulations led us to proceed with the full dataset for all analyses. All post hoc testing was performed using Tukey's Honestly Significant Difference (HSD).

**FIGURE 2:**
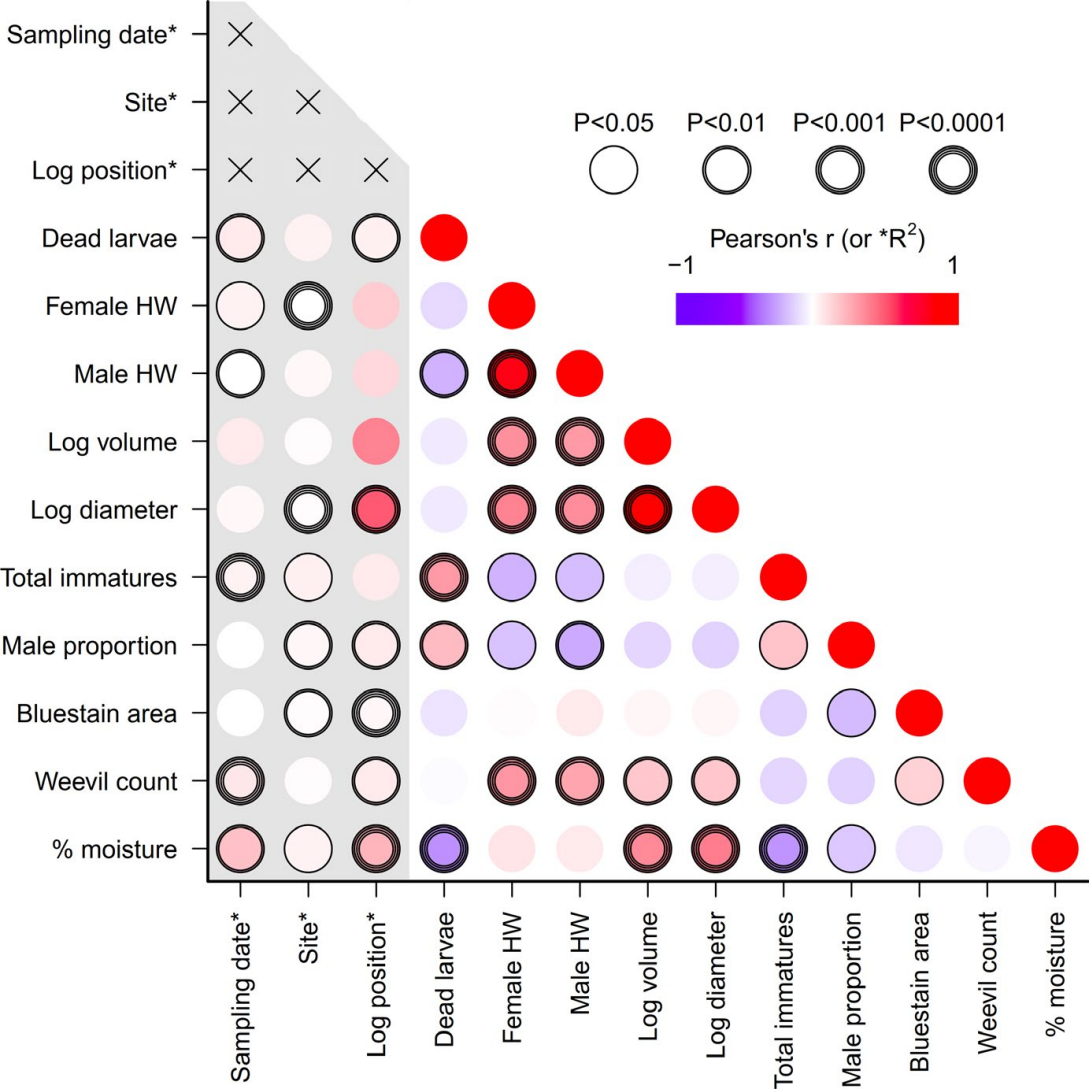
Covariance plot showing the relationship between variables measured at the log scale. Color intensity indicates Pearson's correlation coefficients (r) for continuous variables and *R* ^2^ values from one‐way ANOVAs for categorical variables. Circle width around each dot depicts p‐values (one circle width: *p* ≤ .05; two circle widths: *p* ≤ .01; three circle widths: *p* ≤ .001; four circle widths: *p* ≤ .0001)

Additional predictors were tested as latent variables against residuals from each mixed model described above (i.e., for wasp density, head width, tunnel length, RUE, and male proportion; Table 1a, b, and c) using model selection. As many of the measured variables were correlated to some degree (Figure 2), our models had the potential to suffer from multicollinearity. We dealt with this in two ways. First, testing the importance of environmental variables (e.g., moisture, *Pissodes* count, bluestain area, log diameter, dead larvae) against the residuals of models containing position allowed us to detect additional variation in each primary response variable (larval density, head width, etc.) without violating assumptions. Second, Akaike's information criteria (AIC) were used to compare models and to identify the minimum set of reasonably uncorrelated variables balanced against the explanatory power. Where highly correlated variables (*r* > 0.4) were retained by AIC, all but the strongest correlated predictors were manually removed. We considered all possible models, ranking them by AIC value. Models with ΔAIC values < 2 were considered equivalent (Burnham & Anderson, 2002). For each test, we considered the top five models and reported variables with significant additional explanatory value.

Finally, we calculated survival from early to late larval stage as the change in male and female density between these two time points using the formula (*N*_early_ − *N*_late_)/N_early_. Since sampling was destructive, these densities across time point were calculated using different trees, but since trees were randomly selected from the same sites within the same year (2012), they represent an unbiased estimator of survival across tree position at the site level. We used random resampling (bootstrapping, *n* = 1,000) to produce confidence interval estimates. All nested, mixed models and model selection were performed in JMP 15.0; bootstrapping and graphics were done in R 4.0.2 (R Core Team, 2020).

## 3 RESULTS

We collected and dissected 180 logs and counted and measured 5,775 *S. noctilio* larvae and pupae, including 5,105 males and 670 females (global male:female ratio = 7.6:1). Across sites and collection dates, there was a consistent male bias that increased from bottom to top within trees (from ~5:1 in bottom sections to 8–9:1 in middle and top sections across all time points). Pupae were found in trees only in our September (late) samples. All trees identified as infested contained wasp larvae but not in all sections: *S. noctilio* was absent from 14 bottom sections, three middle sections, and six top sections.

### 3.1 Immature wasp density relationships

**FIGURE 3:**
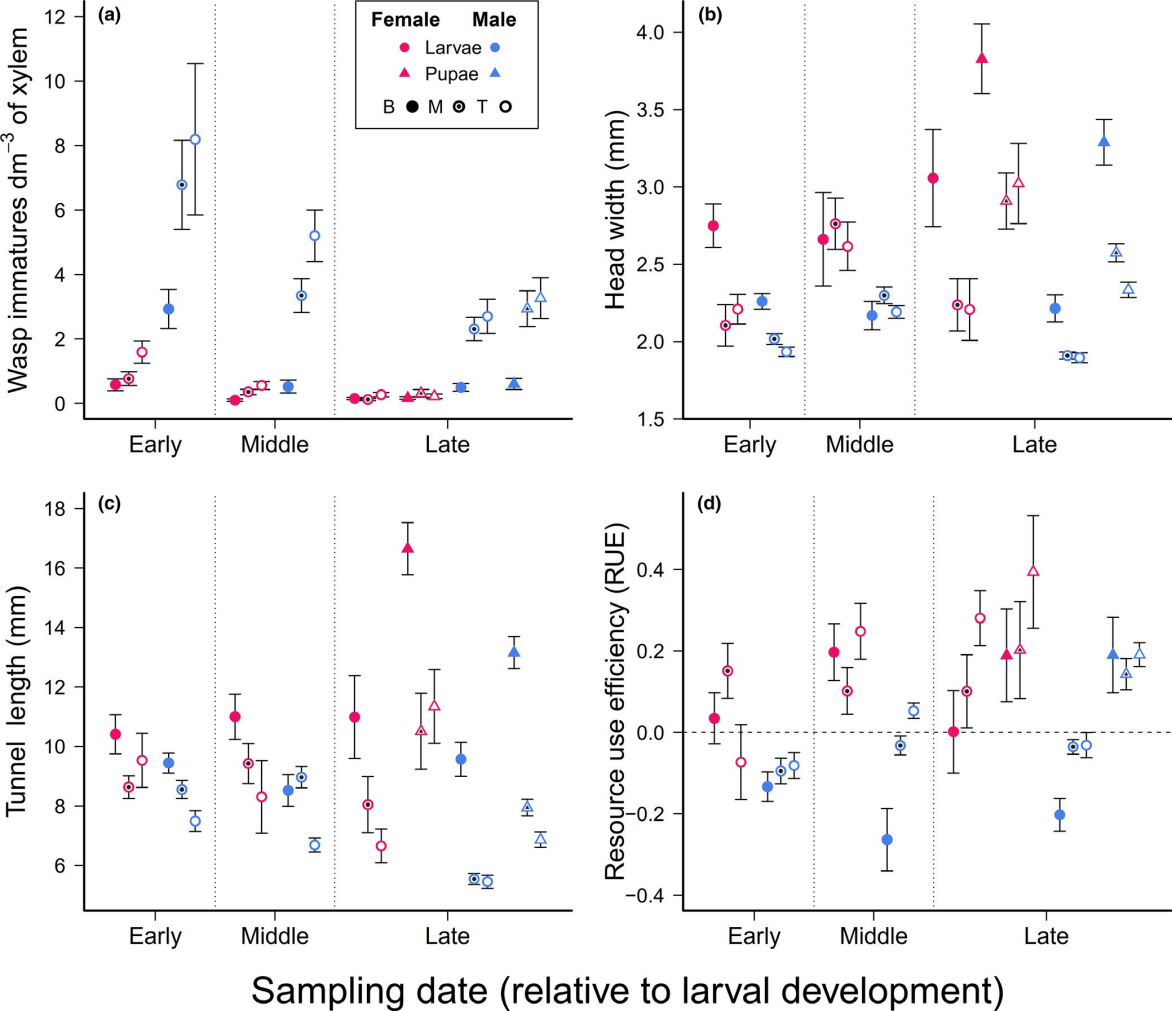
Density of immature wasps dm^−3^ of xylem (a), head width (b), tunnel length (c), and resource use efficiency, or RUE (d) by sampling date, sex, immature stage, and tree position (bottom [B], middle [M], top [T]). Sampling date is listed as early, middle, and late according to when they occurred [in March, June, and September] relative to wasp development within trees. See legend for details. Error bars are *SEM*

Mixed models for wasp density dm^−3^ (log + 1 transformed) across sampling date, log position, and site were strongly predictive (*R* ^2^ = 0.89). Wasp sex was the strongest predictor of larval density (*F* = 272.2 *df* = 1,56; *p* < 0.0001; Figure 3a) followed by the sex × log position interaction (*F* = 45.5 *df* = 2,114; *p* < 0.0001). The sex × position × sampling date was likewise highly significant (*F* = 4.7 *df* = 4,114; *p* < 0.0016). Sampling date showed strong effects on density (*F* = 9.2; *df* = 2,54; *p* = 0.0004). Wasp density declined moderately across sampling dates with June (mid‐development) sampling differing significantly from the March (early) date. Mid‐development sampling densities did not differ significantly from densities at the late sampling date, according to post hoc tests. Log position was also highly significant as a main effect (*F* = 39.2; *df* = 2, 112; *p* = <0.0001) as was site (*F* = 13.2; *df* = 1, 54; *p* = 0.0006). Post hoc tests revealed higher densities of males in the top and middle log sections relative to females of any log position within each sampling date (Tukey's HSD; *α* = 0.05). Likewise, male density in top and middle logs (ranging from 2.6 ± 2.1 and 3.0 ± 2.6 [late sampling] to 6.8 ± 6.2 and 8.2 ± 10.5 [early sampling] for middle and top logs, respectively) was higher than male density in bottom logs (0.6 ± 0.6 to 2.9 ± 2.7), which did not differ from female densities in most log positions, except at the early sampling date. Male density in bottom sections was higher than female densities in bottom sections in the early and middle but not late sampling date.

Random effects of tree(sampling date, site) accounted for negligible variation in wasp density (~0%). Position × tree(sampling date, site) and sex × tree(sampling date, site) accounted for 19.6% and 35.0% of variation, respectively. Random residual variation was 45.4%.

Considering only the late sampling date when pupae were present, sex, log position, and their interaction were all highly significant (*p* < 0.0001 for each; Figure 3a). Wasp density by immature stage (larvae vs. pupae) did not differ markedly (main effect: *F* = 2.5; *df* = 1,146; *p* = 0.12), though this predictor did interact with site (*F* = 15.9; *df* = 1,146; *p* = 0.0001).

### 3.2 *Sirex noctilio* body size relationships

**FIGURE A2:**
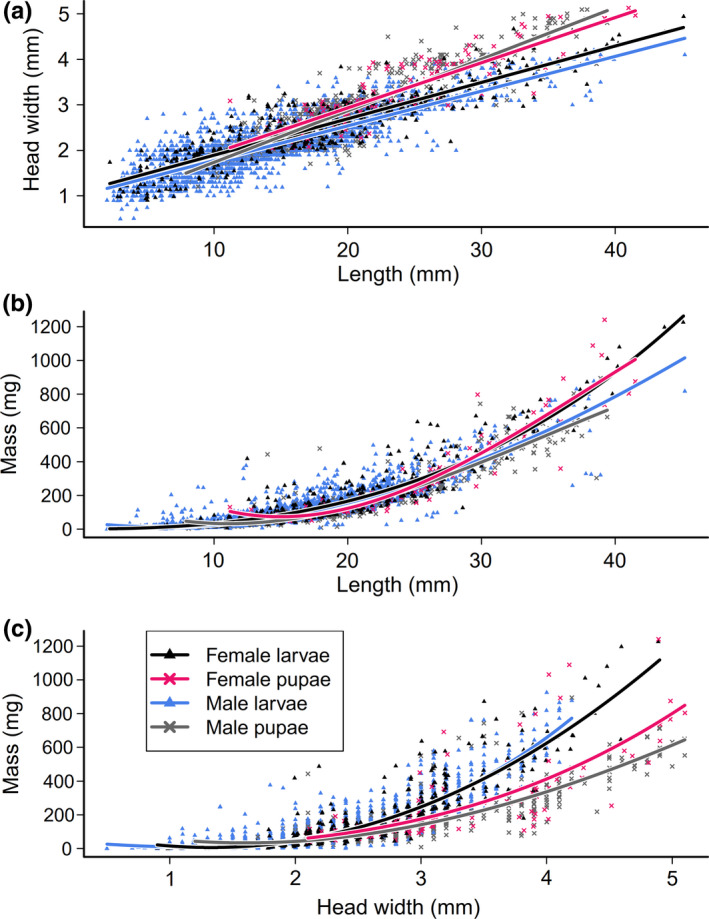
Relationships between body length and head width (a), body length and mass (b), and head width and mass for developing *S. noctilio* larvae and pupae, by wasp sex

Larval and pupal head widths and body length were linearly related (Figure A2a; *F* = 4,973, *df* = 4,5770, *R* ^2^ = 0.78; *p* < 0.0001). Body length‐dry mass and head width‐dry mass relationships were best estimated using quadratic fits (Figure A2b‐c) and were comparable as determinants of body size (*F* = 7,879, *df* = 5,5769, *R* ^2^ = 0.87, *p* < 0.0001 and *F* = 3,529, *df* = 2,5769, *R* ^2^ = 0.75, *p* < 0.0001). For all models, there were significant main effects of sex and immature stage (larvae vs. pupae) on size relationships. Sex × life stage interactions were not significant.

### 3.3 Head width relationships

Nested models for head width where trees were selected randomly to ensure experimental balance (rarefied to six trees per site per sampling date) were substantively similar among each other (*N* = 20 independently randomized subdatasets) and with the full model. In all models, sex and position were highly significant. Eleven of the 20 subdatasets also contained either significant sampling date or sampling date × position effects, all of which were present in the full model, along with marginally significant site × position effects. Both *R* ^2^ values and random effect variance contributions were also roughly similar across all models (*R* ^2^ mean ± *SD* = 0.44 ± 0.04 vs. 0.46 for the full dataset model). As such, for the head width and subsequent analyses, we present only balance‐corrected models based on full datasets. Using head capsule width as the response variable, tree(sampling date, site) accounted for 20% of variation in wasp head width, and log position × tree(sampling date, site), sex × tree(sampling date, site), and sex × position × tree(sampling date, site) contributed 9%, 1%, and 10%, respectively.

Female head width was significantly larger than that of males (*F* = 50.8; *df* = 1,50.7; *p* < 0.0001; Figure 3b). Mean head width declined from the bottom to the top of trees based on post hoc comparisons for both males and females (Tukey's HSD). Head width estimates differed and generally increased from the early to late sampling date, though this relationship was only marginally significant (Tukey's HSD, *p* = 0.05) and was overshadowed by a significant sampling date × position effect (*F* = 7.2; *df* = 4, 94.4; *p* < 0.0001). Our second head width model based solely on the late larval stage (again, the only sampling date when pupae were present) showed strongly significant position (*F* = 53.6; *df* = 2,34.6; *p* < 0.0001), sex (*F* = 42.6; *df* = 1,25.4; *p* < 0.0001), and immature stage main effects (*F* = 67.0; *df* = 1,22.8; *p* < 0.0001) as well as position × stage (*F* = 15.9; *df* = 2,1450; *p* < 0.0001) and position × sex (*F* = 3.7; *df* = 2,613.4; *p* = 0.0252) interactions. Within each log position and instar, female head width was an average of 13.6 ± 5.9% larger than males. Pupal head width (within sex and log position) was 24.6 ± 14.9% larger than for larvae, with the strongest differences in the bottom section (where it was 50% and 35% larger for males and females, respectively). Random effects in this model were dominated by the immature stage × tree(site) interaction responsible for 24% of variation; all other random effects accounted for 3% or less.

Models considering late sampling only were similar, with similar significance profiles for sex and log position among the fixed effects. Here though, wasp stage was also highly significant (*F* = 51.5; *df* = 1, 51.8; *p* < 0.0001) and in its interaction with position (*F* = 7.1; *df* = 2, 315.2; *p* = 0.001).

### 3.4 Tunnel length relationships

Our models of tunnel length were highly significant, with the first model comparing all wasps across sampling points explaining 52% of variation (Figure 3c). Tunnel lengths differed strongly by position within the tree (*F* = 33.9; *df* = 2, 147.5; *p* < 0.0001) and by sex (*F* = 11.7; *df* = 1, 62.7; *p* = 0.0011). The position × sampling date interaction was also highly significant (*F* = 6.6; *df* = 4, 146; *p* < 0.0001). Female tunnels were nearly 14% longer than males (9.6 ± 0.4 vs. 8.4 ± 0.2 mm). Tunnels decreased significantly in length from the bottom to middle and middle to top (Tukey's HSD) and were almost 46% longer in bottom versus top sections. Random effects of tree (sampling date, site) accounted for minimal variation (5.3%) in tunnel length; its interactions with sex and position accounted for an additional 10.6% and 13.6%, respectively. Not surprisingly, the mean length of tunnels containing pupae was longer for those containing larvae (11.0 ± 0.5 vs. 7.6 ± 0.5 mm; immature stage main effect: *F* = 21; *df* = 1, 34.29; *p* < 0.0001). Log position was highly significant (*F* = 50.5; *df* = 2, 175; *p* < 0.0001) with longer tunnels in bottom sections (Tukey's HSD), especially for pupae, as indicated by the immature stage × position interaction (*F* = 16.1; *df* = 1, 1119; *p* < 0.0001). Tunnel length differences did differ between males and females (as above) but similarly for larvae and pupae (*F* = 3.3; *df* = 1, 1010; *p* = 0.0678).

### 3.5 Resource use efficiency (RUE) by log section height, larval sex, and sampling date

Resource use efficiency (RUE = residuals after regressing wasp head width on tunnel length) differed by sampling date (*F* = 4.7; *df* = 2, 75.5; *p* = 0.0119) and sex (*F* = 27.1; *df* = 1, 68.9; *p* < 0.0001). Tukey's HSD supported an increase in RUE from early to late sampling dates, with the middle sampling date exhibiting intermediate RUE that was statistically indistinguishable from either (Figure 3d). Position was only marginally significant as a main effect (*F* = 2.8; *df* = 2,152; *p* = 0.0610) with RUE generally increasing from the bottom to the top of the tree. Position did not appear in any significant interactions with other fixed effects. Overall, the mixed effects model accounted for 39.4% of variation in RUE. The bulk of variance explained in the random effects component was residual variation (72.6%), though the tree × position (sampling date, site) accounted for 14.7% of variation (Wald *p*‐value = 0.013). Tree(sampling date, site) and tree × sex × position (sampling date, site) each contributed approximately 6%.

When considering only the late sampling date, immature stage (larvae vs. pupae) was highly significant (*F* = 32.1; *df* = 1, 815.7; *p* < 0.0001) as was sex (*F* = 9.2; *df* = 1, 62.0; *p* = 0.0035). The fitted model described 32.6% of variation in RUE. Random effects of tree × immature stage(site) and tree × position(site) contributed 15.1% and 9.2% of random variation in this model, with the remainder representing random error variation.

### 3.6 Male proportion by log section height, larval sex, and sampling date

**FIGURE 4:**
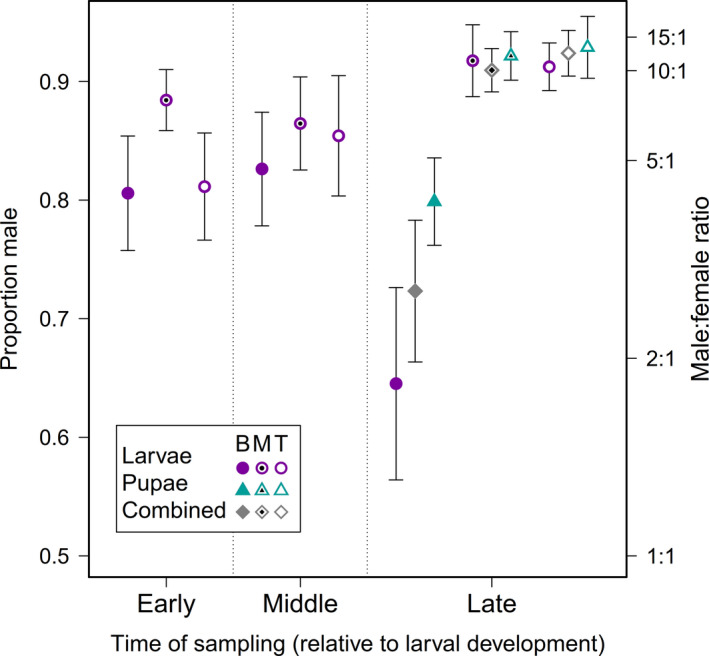
Proportion male (left axis) and male:female ratio (right axis) by sampling date (with early, middle, and late referring each date [March, June, and September] relative to wasp development), sex, immature stage, and tree position (bottom [B], middle [M], top [T]). Error bars are *SEM*

Mixed models accounted for 27.8% of variation in a male proportion (of total larvae) across sampling dates, log positions, sites, and trees. Only position (*F* = 4.5; *df* = 1,49.1; *p* = 0.008) and sampling date × site × position (*F* = 3.0; *df* = 4, 93.6; *p* = 0.022) were significant as fixed effects. As a random effect, tree(sampling date, site) only accounted for just over 3% in variation in male proportion. Across sites, position, and sampling date, male proportion ranged from 72%–92% (mean ± *SD* = 84.4 ± 0.02%) of all larvae and pupae combined (Figure 4). Pupae were only very slightly and nonsignificantly more likely to be male. Post hoc tests revealed that male proportion was lowest in bottom logs and similar in middle and top logs.

### 3.7 Survival rates by log section height, larval sex, and sampling date

Random sampling from the same sites during early and late stages of larval development within the same season allow the estimation of log position and sex‐specific survival rates from small larvae to large larvae and pupae. Survival estimates were variable when bootstrapped within sites, in some cases exceeding 100% in certain log section‐sex combinations (Table 2). When sites were pooled, however, there was a general trend of higher survivorship for females in bottom (58.3 ± 18.1%) and middle tree sections (59.4 ± 17.9%) relative to top sections (32.5 ± 8.5%). This pattern was generally reversed for males where survival estimates were high in the top (76.5 ± 8.5%) and middle sections (78.6 ± 18.0%) but moderately low in bottom sections (35.4 ± 9.2%). Pupation rates likewise differed by sex and log section, with the highest rates for males again in the top and middle sections (59.4 ± 17.9% and 32.5 ± 8.5%) and lowest in the bottom (21.4 ± 5.6). For females, pupation was highest in the middle section (43.6 ± 16.3%) and lowest at the top (14.5 ± 3.8%).

**TABLE 2:** Survival and pupation rates from early to late sampling dates, % (±*SD*)

Log position	Survival rate (%)		Pupation rate (%)	
	Females	Males	Females	Males
Bottom	58.3 (±18.1)	38.4 (±9.2)	30.8 (±10.9)	21.4 (±5.6)
Middle	59.4 (±17.9)	78.6 (±10.8)	43.6 (±6.3)	44.1 (±6.2)
Top	32.5 (±8.5)	76.5 (±18.0)	14.5 (±3.8)	41.3 (±8.9)

Note

Error estimates based on 500 bootstrapped samples (selecting *n* = 14 trees per sample).

### 3.8 Biotic and abiotic drivers of observed variation

Latent variable contribution to residuals of wasp density revealed seven models of equivalent quality according to AIC (ΔAIC < 2) containing between two and four parameters and explaining between 10.2% and 11.1% of residual variation in wasp density. All contained different permutations of log moisture, bluestain area, log diameter, and density of dead larvae. The most preferred model contained both log moisture and bluestain cross‐sectional area, both of which were negatively and linearly associated with residual variation in density (parameter estimates: −0.017 ± 0.003 and −0.018 ± 0.003, respectively). Results were qualitatively similar for the late sampling date modeling larvae and pupae.

Four equivalent models (ΔAIC < 2) were identified as predicting mean head width residuals, each containing a combination of one to three parameters and with *R* ^2^ values ranging from 0.112 to 0.114. Top parameters were log moisture, count of dead larvae per log, and *Pissodes* sp. density. The top model explaining 11.3% of variation in head width model residuals contained log moisture, *Pissodes*, and dead wasp density. Moisture (slope = −0.02; *F* = 8.1; *df* = 1, 153; *p* = 0.0051) and dead larval count (slope = −0.02; *F* = 8.3; *df* = 1, 153; *p* = 0.0046) were negatively associated with mean residual head width variation, while *Pissodes* sp. count was positively associated (slope = 0.004; *F* = 6.8; *df* = 1, 153; *p* = 0.0098).

Five equivalent models were identified as the best predictors of residual variation in tunnel length with *R* ^2^ values ranging from 0.06 to 0.08. The top model included log moisture and the count of dead larvae, each of which was negatively associated with tunnel length (parameter estimates were −0.01 ± 0.005 for each). While both were highly significant, the two predictors combined described only 6.1% of variation. Log diameter showed in several of the top models and was positively associated with tunnel length residuals.

Model selection identified five equivalent models (ΔAIC < 2) predicting mean residual variation in RUE (*R* ^2^ value ranging from 0.08 to 0.09). Most top models contained wood volume (or the highly correlated log diameter) and *Pissodes* density, dead wasp larvae, and moisture. The top model containing significant predictors explained 8.5% of variation and contained wood volume (parameter estimate = −0.00004; *F* = 11.6; *df* = 1, 152; *p* = 0.001) and *Pissodes* density (parameter estimate = 0.004; *F* = 6.9; *df* = 1, 152; *p* = 0.009).

## 4 DISCUSSION

Native and invasive populations of *S. noctilio* around the world exhibit huge size variation among individuals, well in excess of what is typically seen in insects (Madden, 1981; Neumann et al., 1987; Corley et al., 2007; Hurley et al., 2008; Krivak‐Tetley et al., In review). High size variation was confirmed by our detailed study of *S. noctilio* larvae and pupae in South Africa as well as for adults (Hurley et al., unpublished data). Likewise, South African populations of this wasp exhibit strongly male biased sex ratios ranging from 10 to 15:1 in newly infested areas but typically (though not in all cases) decrease to 3 or 4:1 within a few years (Tribe & Cillié, 2004). We studied patterns of density, larval size, tunnel length, and male proportion at two sites in the Mpumalanga Province of South Africa. We examined how this variation might be attributable to differences in larval environments within and among trees.

Our data show strong support for differences in all measured responses by log position within the tree. For example, although bottom sections contain the fewest *S. noctilio*, larvae and pupae are nearly always the largest in bottom tree positions and the smallest in the top positions. Top positions represent the greatest contribution to *S. noctilio* population numbers in South Africa (and in North America; Krivak‐Tetley et al., 2020), but contain a disproportionate number of males and smaller individuals overall (i.e., females were 22% smaller, and males were 14% smaller in top sections compared to bottom sections). Based on this and other work (Hurley et al., 2008; Ryan et al., 2012), trees are clearly not uniform environments from top to bottom from the perspective of *S. noctilio*. The reasons for differential quality as a resource from the bottoms to the tops of trees are not entirely clear but could include factors such as bark thickness, water potential, drying rate, wood density and volume, as well as susceptibility to other insects and fungi whose distributions within trees may also reflect microclimate preferences and/or life history or behavior (Chow & Obermajer, 2007; Domec & Gartner, 2001; Fox et al., 1990), with direct or indirect consequences for *S. noctilio*. Our data are consistent with previous studies which have found that *S. noctilio* emergence rate, size, and parasitism rate vary significantly by positions within tree and also that water potential differences may influence *A. areolatum* growth, with the potential to adversely affect the food source for feeding larvae (Hurley, Hatting, et al., 2012; Long et al., 2009).

Not surprisingly, sampling date was a significant predictor in many of our analyses. This is expected for head width as larvae grow and develop during the year. However, strong interaction effects with wasp density, head width, tunnel length, RUE, and male proportion were evident when sampling date was crossed with tree position, sex, and site. Differential growth and survival in wasp immatures over time thus depend on both intrinsic (e.g., larval or pupal sex) and extrinsic factors (e.g., tree section, microenvironmental variation, and densities of co‐occurring biota). Both male and female densities decreased from the early to late sampling date, but survival rates were highly dependent on tree position. For example, male bias in middle and top sections was ~5.6:1 within approximately 2 months after oviposition (early) but increased to 11.1:1 approximately 9 months after oviposition (late sampling date). However, there was an opposite effect in bottom positions as male survival was significantly lower than in other sections, and well below that of females in bottom logs (early stage M:F 4.1:1, late stage M:F 2.6:1). This finding suggests two factors of relevance to *S. noctilio* populations: (a) that some larval environments favor males while others favor females; and (b) that ovipositing females do not effectively optimize the placement of fertilized versus unfertilized eggs based on growth and survival probabilities, at least in the invasive population studied. This stands in contrast to other xylophagous hymenopterans (sawflies) that adjust the placement of male versus female eggs in response to host quality (Cárcamo et al., 2005; Craig et al., 1992; Mopper & Whitham, 1992; Morrill et al., 2000). This finding also complements recent work showing that genetic drivers of male bias (specifically low genetic diversity together with a complementary sex determination system) are overshadowed by ecological factors (Queffelec et al., 2019). Higher female survival in bottom logs could reflect higher resource quality which may be more important for females who must invest in egg production as well as growth. However, this would not explain lower male survival in bottom relative to other sections.

RUE varied strongly by sampling date and by tree position via its interaction with sampling date. There were only small, marginally significant differences by sex. The general trend of decreasing efficiency from the early/middle to late time point could suggest that larval become more mobile (relative to growth gains) as they approach completion of development. There are a number of factors that could potentially be linked to RUE, but aspects of xylem density or nitrogen content, water availability, and the competitive environment with other microbes are among the most likely to influence growth of *A. areolatum* (as well as other microbial mutualists) and therefore the development time for *S. noctilio* (Hurley, Hatting, et al., 2012; Ryan et al., 2012). In our dataset, RUE was significantly influenced by moisture and the density of *Pissodes* sp., but bluestain area showed no significant trend.

Tunneling behavior can reflect ecological or adaptive (or nonadaptive) responses beyond the efficiency of nutrient capture (e.g., avoidance of natural enemies or competition; Djemai et al., 2000). *Sirex noctilio* has been shown to not pass xylem fragments through its gut; rather, mouthpart morphology is more suggestive of a feeding strategy where wood fragments are squeezed (probably as a method of extracting polysaccharides) and are then passed under the body and packed by the advancing larvae (Thompson et al., 2014). Additionally tunnels extend well outside the area of xylem colonized by *A. areolatum*—a source of key digestive enzymes perhaps as a strategy to avoid parasitism by *D. siricidicola* which feeds on the fungus prior to encountering its host. Still, understanding size‐ and stage‐specific patterns in tunneling distance is important to understanding when and under what developmental or ecological (see “Additional predictors” below) conditions larvae move and grow.

Overall, only a marginal amount of variation in all of the response variables considered was accounted for by the random variable tree (between 0% and 15%). This suggests that relevant differences in resource quality probably occur more strongly within trees. Stated differently, to some degree, all trees, once attacked, are of similar quality as a food source and habitat. Position within the tree (and to a lesser degree, microsite variation within each position) is more important than among tree variation for developing larvae. For example, the tree × position random effect (nested within sampling date and site) often accounted for considerably more variation than tree itself (between 0% and 22%), and the interaction effect of tree × sex (also nested within sampling date and site) accounted for moderate variation (between 7% and 14%). Since all of the trees selected for this study were successfully attacked by *S. noctilio* and harbored live and developing larvae and/or pupae, variability that may have influenced oviposition choice and/or egg survival (and/or our ability to detect attack) was not included in our analyses.

In a recent study of *S. noctilio* in North America, adult size was influenced at least in part by abiotic conditions, including wood moisture tree host, height, and intraspecific density (Foelker, 2016). Other plausible environmental factors that may influence adult size include tree defenses, the identity and abundance of co‐occurring insect or fungal species (including bluestain fungi which putatively compete with *S. noctilio's* own fungal symbiont; Foelker, 2016), nematode parasitism or other sublethal infection (Haavik et al., 2016; Villacide & Corley, 2008), degree day accumulation, and/or duration of the larval period. The current study demonstrates that the local xylem environment, particularly as it differs across tree sections, drives a considerable proportion of size variation in South Africa.

The ecological determinants of sex ratio—including those that are likely to covary with invasive status—are likewise numerous and complex. Females of some species, including within the Hymenoptera, show a preference for placing unfertilized eggs (which will develop into smaller, more evolutionarily expendable males) in resource‐poor environments (Craig et al., 1992; Morrill et al., 2000). Poor mating success leading to an overabundance of unmated females (which can only produce males) is also a possibility, as is the overproduction of diploid males in populations with low genetic diversity where sex is determined by a complementary sex determination system (Boer et al., 2012; Cook & Crozier, 1995). Queffelec et al. (2019) recently explored several proposed mechanisms of male bias in South African populations of *S. noctilio* using field data and simulation modeling. While this study found a relatively high proportion of unmated females (39%), the authors ultimately concluded that female investment in males (among mated females) is likely a stronger driver of male bias at the egg stage. Facultative sex ratio shifts in response to host quality have been observed in several insect populations, including Hymenoptera (e.g., Euura sawflies; Craig et al., 1992). Female control over offspring sex appears to be common in the *S. noctilio* system, as significant male bias was already present at the early sampling date and varied consistently by log section. Our study additionally shows that larval survival postoviposition is a major driver of male bias.

Interestingly, biotic agents had only modest influence on pattern in woodwasp size, density, or survival. The density of *Pissodes* sp. was among the top predictors explaining residual variation in a number of our modeled parameter, but its contribution was never greater than 10%. Bluestain cross‐sectional area was likewise a weak predictor of larval success. This contrasts with other studies in Spain and North America that find stronger support for resource competition or other antagonistic interactions between *S. noctilio and* bluestain fungi, which appear to inhibit wasp success (Haavik et al., 2015; Lombardero et al., 2016).

## 5 CONCLUSIONS AND IMPLICATIONS FOR MANAGEMENT

This study sheds light on some of the drivers of size and sex ratio variability in *S. noctilio*, and it suggests that postzygotic mechanisms (i.e., ecological effects on larvae and pupae within trees) explain significant variation in both. Based on our results, the impressive size variation and male bias in South African *S. noctilio* populations arise in no small part from variation within and among trees (primarily the former, at least within *P. patula*). In comparison to mating limitation and other genetic mechanisms with the potential to influence sex ratio (Queffelec et al., 2019), ecological effects linked to microsite variation within trees appears strong. The observed male bias in South Africa could emerge as a consequence of introduction into evolutionarily “unfamiliar” conditions, and the tendency for male proportion to decrease over time may therefore reflect adaptation to the local resource environment, either in reproductive investment by females, in larval development, or both. In addition, a more nuanced understanding of how male and female *S. noctilio* offspring are partitioned, survive, and develop within and among trees has several implications for management. For example, the current practice of augmentative releases of parasitic nematodes in South Africa focuses primarily on the lower trees bole, because of higher parasitism rates obtained in that section and because of the ease of inoculating standing trees, as compared to felled trees if the middle and top section were also inoculated (Hurley, Croft, et al., 2012). Our findings generally support this approach because female wasps, responsible for transmitting parasitic nematodes, disproportionally emerge from those sections. However, increased focus on middle sections may be warranted, given higher female pupal densities and higher survivorship there. Inoculation of small, strongly suppressed trees that tend to dry out quickly may not effectively target large females.

## CONFLICT OF INTEREST

The authors declare no competing interests.

## AUTHOR CONTRIBUTIONS

**Jeff R. Garnas:** Conceptualization (lead); data curation (supporting); formal analysis (lead); funding acquisition (lead); investigation (equal); methodology (equal); project administration (lead); supervision (lead); validation (lead); visualization (lead); writing – original draft (equal); writing – review and editing (lead). **Katie E. Vann:** Formal analysis (supporting); investigation (supporting); methodology (equal); visualization (supporting); writing – original draft (supporting). **Brett P. Hurley:** Conceptualization (supporting); methodology (supporting); supervision (supporting); writing – review and editing (supporting).

## Data Availability

The data for this study are openly available in Dryad at http://www.datadryad.org using the following https://doi.org/10.5061/dryad.dncjsxkxh.
